# Time-to-death and its predictors among under five children in the developing regions of ethiopia: a cross sectional study

**DOI:** 10.1186/s12887-025-06215-1

**Published:** 2025-11-05

**Authors:** Gebru Gebremeskel Gebrerufael, Brhane Gebrehiwot Welegebrial, Mehari Gebre Teklezgi, Zeytu Gashaw Asfaw, Yemane Hailu Fissuh, Gebregewergis Alemu Gebremedhn

**Affiliations:** 1 Department of Statistics, College of Natural Science, Adigrat University, Adigrat, Ethiopia; 2 Department of Pharmacy, College of Medicine and Health Sciences, Adigrat University, Adigrat, Ethiopia; 3 Department of Preventive Medicine, College of Health Sciences, Addis Ababa University, Addis Ababa, Ethiopia; 4 Department of Statistics, College of Natural Science, Aksum University, Aksum, Ethiopia

**Keywords:** Ethiopia, Cox PH regression, Under-five children mortality, Predictors

## Abstract

**Background:**

The under-five children mortality rate (U5CM) is still a global public health concern, especially in Ethiopia and other countries in Sub-Saharan Africa (SSA). Unfortunately, the issue is notably underestimated and underreported, making it difficult to fully assess the severity of the crisis in the nation-state’s emerging regions. Regrettably, no research has been done to determine the time-to-death and its predictors for children under five in the developing regions of Ethiopia. Thus, the aim of this study was to determine the time-to-death and its predictor factors among children under five years old in the developing regions of Ethiopia from March 21, 2019, to June 28, 2019.

**Methods:**

This is a secondary analysis of data collected in a cross-sectional study that was done among under-five children in the developing regions of Ethiopia between March 21, 2019, and June 28, 2019. The Kaplan-Meier (K-M) survival curve was utilized to display the statistically significant variance across categorical variables, and the survival time was evaluated using the log-rank test. The Cox Proportional Hazards (PH) regression model analysis of bivariable and multivariable variables was fitted to identify the predictor factors of time-to-death. The investigation’s findings were presented utilizing tables, text, graphs, and charts. The degree of significance was determined using an adjusted hazard ratio (AHR) with a 95% confidence interval (CI) and a p-value less than 0.05.

**Results:**

In the study, a total of 2,019 children under five were included. The overall under-five child mortality rate in developing regions of Ethiopia was 8.1% (95% CI 7.0%, 9.4%). In the multivariable Cox PH regression model analysis, multiple birth type (AHR: 2.9, 95% CI: 1.34, 6.46), mothers being AntiNatal Care (ANC) follow-up (AHR: 2.0; 95% CI: 1.08, 3.87), not initiating exclusive breastfeeding (AHR: 2.7, 95% CI: 1.23, 5.78), the female sex of the child (AHR: 0.56, 95% CI: 0.333, 0.934), and the head of household being female (AHR: 0.47, 95% CI: 0.236, 0.923) were recognized as main predictors of time-to-death among under five-children.

**Conclusions:**

The study highlights an important under-five child mortality rate of 8.1% in the developing regions of Ethiopia. Important predictors identified through multivariable Cox Proportional (PH) regression model analysis include multiple birth type, lack of mother’s antenatal care (ANC) follow-up, the child’s sex, gender of household head, and failure to begin exclusive breastfeeding. Therefore, in order to decrease the high rate of mortality among children under five, the government should emphasize improvement of the ANC services, promote exclusive breastfeeding, make a targeted intervention for multiple births, and support female households in the developing regions of Ethiopia.

## Background

The Under-five Children Mortality (U5CM) is defined as the likelihood of dying between birth and exactly five years of age, expressed per 1,000 total live births [1]. The U5CM is one of the most important indices of social and national progress since it reflects health fairness and access [2]. The first five years of life are the most critical period for a child’s physical and mental development [3].

The U5CM is still to be a serious global public health concern, especially in nations low-and middle-income countries like Africa and Sub-Saharan Africa (SSA) [4, 5].

Although there is a worldwide reduction in under-five children mortality, from 5.9 million deaths in 2015 to 5.3 million in 2018, still there is a high U5CM rate in African nations (81 deaths per 1,000 live births), comprising Ethiopia, which is approximately seven times greater than that of European countries [3, 4]. In Africa, the overall U5CM rate was 37.6 per 1,000 total live births and in this region U5M rate contributes to 14% of the worldwide load of child death and mainly, in sub-Saharan Africa (SSA); it accounts for approximately 62% of U5CM [1, X21].

Moreover, many SSA countries experienced the greatest U5CM suffering, with one in every thirteen live births disappearing before celebrating five years old, a fifteen-fold increase over developing countries. In 2018, half of all these mortalities occurred in five countries: Nigeria, India, Ethiopia, Pakistan, and the Democratic Republic of the Congo [5].

For the majority of middle-income and developing nations, the fourth Millennium Development Goal (MDG) was an incomplete program intended to reduce the U5M rate by two thirds (2/3) [6].

One of the most pressing issues that need to be prioritized for U5CM is Ethiopia [5]. Therefore, Ethiopia needs further investigation in order to solve such a kind of problem. To solve such a problem, Ethiopia is proposed to reduce the U5CM to ≤ 25 deaths per 1000 total live births by 2030 [7]– [8]. However, research indicates that there is a significant regional variation in the U5CM rates, and Ethiopia continues to rank among the countries with the worst public health issues [5]. According to the Ethiopian Mini Demographic and Health Survey (EMDHS) 2019 report, U5CM rate per 1,000 total live births in Somali, Benishangul-Gumuz, Gambela, and Afar were 101, 90, 86, and 76, respectively [9]. This suggests that the U5CM rate is still extremely high in some of Ethiopia’s developing regions. Predictor factors of under-five child mortality include breastfeeding status, child sex, antenatal care follow-up, and birth type, according to findings from prior different literatures [10–13].

Ethiopia is attempting to lower the U5CM rate; however, there is still a significant increase in the developing regions of Somalia, Benishangul-Gumuz, Gambela, and Afar [9]. This is despite efforts to improve coverage, quality, and utilization of skilled care, critical newborn care, and management of preterm and low birth weight. Therefore, this study aimed to explore the predictor factors of U5CM in these developing regions of Ethiopia. This study may raise awareness among the public, medical professionals, and politicians about appropriate interventions to decrease the U5CM rate.

## Materials and methods

### Study settings and data source

The investigation used the 2019 Ethiopian Demographic and Health Survey (EMDHS) datasets, which were found using a community-based retrospective cross-sectional study design. The central statistics agency office [6] has been using nationally representative household survey data that is collected every five years with the primary goal of providing current estimates of important indicators of health and demographic characteristics. Ethiopia is divided administratively into two major cities, Addis Ababa and Dire dawa, and nine regions: Amhara, Oromia, Benishangul-Gumuz, Gambela, Afar, South Nation Nationalities and Peoples’ Region, Harari, and Somali. Once more, these areas are divided into developed and developing regions. The majorities of the emerging regions are home to disperse pastoralists and are called Gambela, Benishangul-Gumuz, and Somali. Developing regions are often characterized by inadequate infrastructure, limited access to health services, poverty, drought, and a lack of comprehensive and well-defined rules [14]. The Tigray, Amhara, Oromia, SNNPR, and Harari areas are developed, as are their city governments. These regions are distinguished by a relatively dense population, improved infrastructure, and easy access to services for healthcare and education.

### Sampling techniques

The 2019 EMDHS utilized two stages of stratification and selection of a sample. To create 21 sampling strata, each region was divided into rural and urban areas. Two phases of independent selection were used to choose the sample of enumeration regions in each stratum. A total of 305 enumeration areas—212 in rural and 93 in urban areas—were chosen in the first stage using independent selection in each sample stratum and a probability proportionate to the size of the enumeration area. The obtained household lists were used as a sampling frame in the second stage of household selection.

In the second step, the newly formed household listing is used to select a fixed number of 30 homes per cluster, with each cluster having an equal chance of being chosen in a methodical manner. Interviews were available for all reproductive women between the ages of 15 and 49 who were either guests who spent the night before the survey or permanent residents of the chosen households. The study covered children born in developing regions of Ethiopia no more than five years before the survey. For analysis, 2,019 children under the age of five were included. The whole EMDHS 2019 report [9] included information on the sampling process as well as the total data collection.

## Study variables

### Outcome variable

The dependent variable (time-to-death) was the age of a child (in months) when she/he died after live birth (the commencement time). Similarly, events (uncensored) refer to children who died either before or at the time of the EMDHS data collection period. In contrast, “censored” is defined as an occurrence (children alive) that occurred after the last date of EMDHS data collection [15].

### Predictor variables

Based on the previously conducted research, various demographic, socio-economic, and environmental factors were included as predictor variables presented in Table 1.

**Table 1:** Operational definition and categorization of associated variables used in the study

Variables	Categorizations of independent variables
Sex of child	Sex of child (0 = male, 1 = female)
Maternal age	Maternal age (in years) (0 = 45–49, 1 = 40–44, 2 = 35–39, 3 = 30–34, 4 = 29 and below)
Toilet facility	Type toilet facility (0 = yes, 1 = no)
Sex of household head	Sex of household head in the family (0 = male, 1 = female)
Parity	Total children ever born (0 = 3 and below, 1 = 4–5, 2 = 6–8, 3 = 9 and above)
Birth order number	Birth order number (0 = 3 and below, 1 = 4–5, 2 = 6 and above)
Birth type	Birth type pregnancy (0 = singleton, 1 = multiple)
Breastfeeding	Current breastfeeding (0 = no, 1 = yes)
ANC follow up	Antenatal care follow up (0 = yes, 1 = no)
Region	Type of region (1 = Afar, 2 = Benishangul-Gumuz, 2 = Somalia, 3 = Gambela)
Maternal educational level	Maternal educational level (0 = higher, 1 = secondary, 2 = primary, 3 = no education)
Media exposure access	Media exposure access (0 = yes, 1 = no)
Wealth index	Wealth index combined of households (0 = rich, 1 = middle, 2 = poor)
Currently pregnant	Currently pregnant (0 = no, 1 = yes)
Current contraceptive method	Current contraceptive method (0 = no, 1 = yes)
Marital status	Mothers’ marital status (0 = married, 1 = separated, 2 = widowed, 3 = Divorced)
Place of delivery	Place of delivery (0 = with health facility, 1 = home)
Delivery by caesarean section	Delivery by caesarean Sect. (0 = no, 1 = yes)
Counseling during pregnancy	Counseling during pregnancy (0 = yes, 1 = no)

### Data management and analysis

In this study, STATA version 14 statistical software was used to clean, code, and analyze the gathered data after they were imported into Excel in CSV format. Descriptive statistics, such as percentages and frequency distributions, were employed in the study to define the sample data. Under five-year-old children’s survival probabilities were compared using the Kaplan-Meier (K-M) non-parametric survival curve technique. The Cox PH model is one of the most used kinds of regression models in survival analysis. Moreover, this model has an important role in the investigation of the survival time-to-death of under-five children by estimating hazard ratios (HRs) and 95% confidence intervals (CIs). The general Cox PH regression model can be defined as;

1 $$\begin{aligned} \:{h}_{i}\left(t\right)=&{h}_{0}\left(t\right)*exp\left({{\upbeta\:}}_{1}{\text{w}}_{i1}+\:{{\upbeta\:}}_{2}{\text{w}}_{i2}\:+\:{{\upbeta\:}}_{3}{\text{w}}_{i3}\right. \\& \left.+\:{{\upbeta\:}}_{4}{\text{w}}_{i4}+\dots\:\dots\:+{{\upbeta\:}}_{P}{\text{w}}_{iP}\right) \end{aligned}$$

$$\begin{aligned} \:\text{l}\text{o}\text{g}\left({h}_{i}\left(t\right)\right)=&{\text{l}\text{o}\text{g}[h}_{0}\left(t\right)*exp\left({{\upbeta\:}}_{1}{\text{w}}_{i1}+\:{{\upbeta\:}}_{2}{\text{w}}_{i2}\:+\:{{\upbeta\:}}_{3}{\text{w}}_{i3}\right. \\& \left.+\:{{\upbeta\:}}_{4}{\text{w}}_{i4}+\dots\:\dots\:+{{\upbeta\:}}_{P}{\text{w}}_{iP}\right)]\:\:\:\:\:\:\:\:\: \end{aligned}$$

$$\begin{aligned} \:=&{{\upbeta\:}}_{0}+\left({{\upbeta\:}}_{1}{\text{w}}_{i1}+\:{{\upbeta\:}}_{2}{\text{w}}_{i2}\:+\:{{\upbeta\:}}_{3}{\text{w}}_{i3}\right. \\& \left.+\:{{\upbeta\:}}_{4}{\text{w}}_{i4}+\dots\:\dots\:+{{\upbeta\:}}_{P}{\text{w}}_{iP}\right).\:\:\end{aligned}$$

This model was used to investigate and to check the impact of each independent variable on the mortality rate. Where; $\:{h}_{i}\left(t\right)\:$ is denotes as the hazard for an event for patient i at time t is determined by a set of p covariates ($\:{\text{w}}_{i1}$, $\:{\text{w}}_{i2}$, $\:{\text{w}}_{i3}$, $\:{\text{w}}_{i4}$……, $\:{\text{w}}_{ip}$), whose impact is measured by the size of the respective coefficients ($\:{{\upbeta\:}}_{1}$, $\:{{\upbeta\:}}_{2}$, $\:{{\upbeta\:}}_{3}$, $\:{{\upbeta\:}}_{4}$……, $\:{{\upbeta\:}}_{p}$). The term $\:{h}_{0}\left(t\right)$ is denotes the baseline hazard for a mortality [16]– [17]. The proportionality assumptions of Cox PH regression model analysis were assured on each predictor variable and on the global test of proportionality. After the PH assumption had been checked, the bivariable Cox PH regression model was fitted for each predictor variable. Crude and adjusted hazard ratios with a 95% confidence interval (CI) were used to measure the strength of association and to explore statistically significant predictors. In the multivariable Cox PH regression model analysis, variables having P-value < 0.05 were found as significant predictors of mortality in under-five children. The goodness of fit of the final model was checked by the Likelihood Ratio Test (LRT).

## Results

### Descriptive statistics

In this study, a total sample of 2,019 under-five children was included. The majority (56.32%) of under-five children were born to mothers with no formal education, and 1,085 (53.7%) of under-five children were males. Of the total under-five children, 1,656 (82.02%) of their mothers didn’t have media exposure access, and 1,194 (59.14%) of under-five children were born to mothers aged 29 and below. The majority (96.09%) of under-five children were singleton births. About 1,135 (56.22%) of the mothers had not had ANC follow-ups during their pregnancy (Table 2).

**Table 2:** Test of proportional-hazards assumption (STATA version 14)

Predictor variables	Rho	Chisq	Df	Prob > Chisq
Sex of child (ref. = male)				
Female	0.0094	0.1	1	0.939
Maternal age (ref. = 45–49)				
40–44	0.05328	0.19	1	0.663
35–39	−0.09738	0.5	1	0.481
30–34	0.04818	0.16	1	0.691
29 and below	0.05872	0.21	1	0.646
Toilet facility (ref. = yes)				
No	−0.03769	0.1	1	0.756
Birth type (ref. = single)				
Multiple	−0.05534	0.2	1	0.651
Sex of household head (ref. = male)				
Female	−0.05644	0.2	1	0.652
Parity (ref. = 3 and below)				
4–5	−0.08667	0.5	1	0.486
6–8	−0.11359	0.91	1	0.348
9 and above	−0.13054	1.15	1	0.284
Birth order (ref. = 3 and below)				
4–5	−0.01435	0.01	1	0.912
6 and above	0.09539	0.54	1	0.462
Breastfeeding (ref. = no)				
Yes	0.10489	0.82	1	0.365
ANC follow up (ref. = yes)				
No	0.19313	2.63	1	0.105
Global test		11.75	15	0.698
N.B: - chisq refers to chi-square statistic value, DF refers to degree of freedom, Prob > Chisq refers to P-value				

**Fig. 1:**
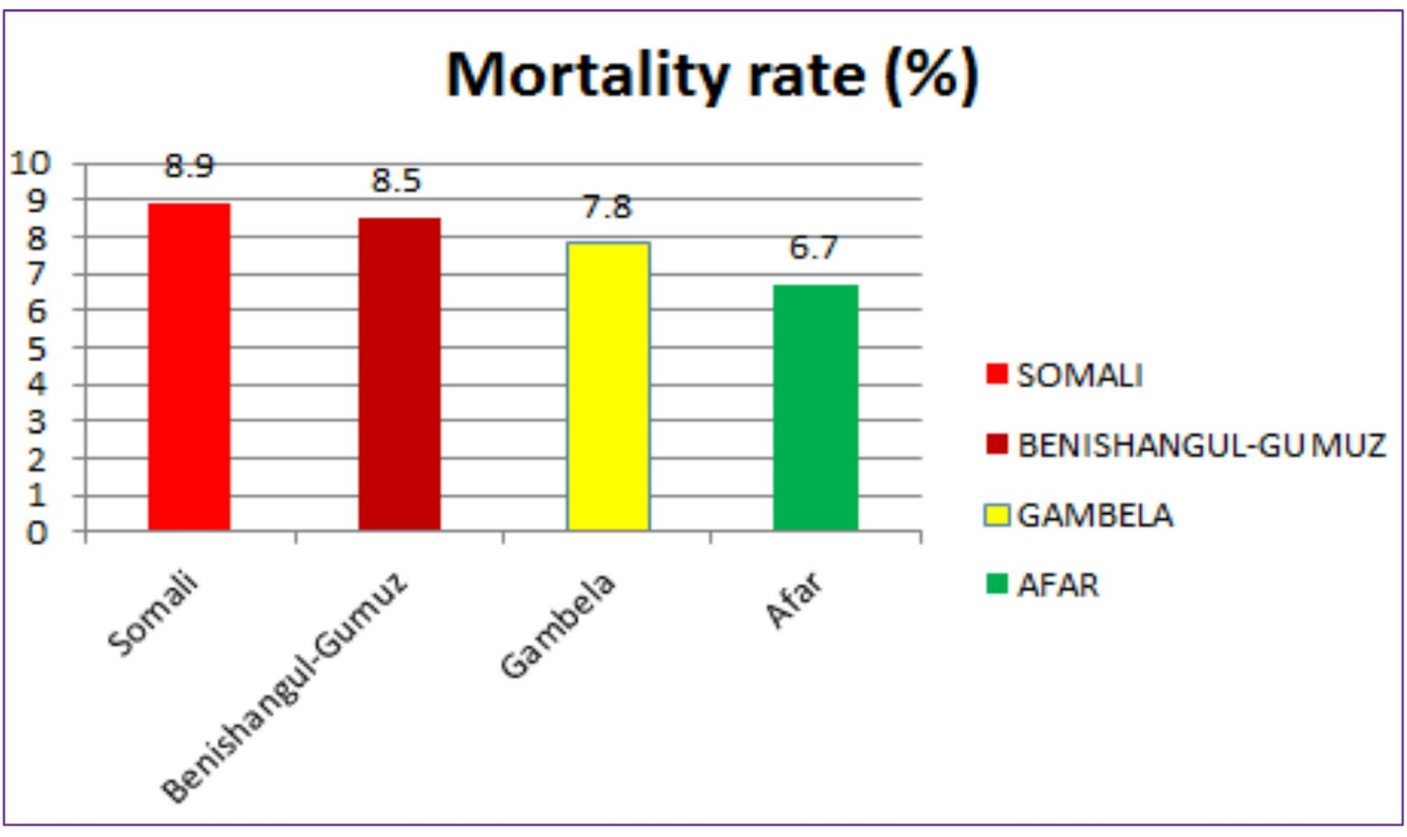
The under-five children mortality rates in developing regions of Ethiopia, 2019

The under-five child mortality rate in developing regions of Ethiopia was 8.1% (95% CI 7.0%, 9.4%) per 100 total live births, which was highest in the Somali region (8.9 per 100 live births) and lowest in the Afar region (6.7 per 100 total live births) (see Fig. 1).

### Comparison of the survival ability of under-five children

**Fig. 2:**
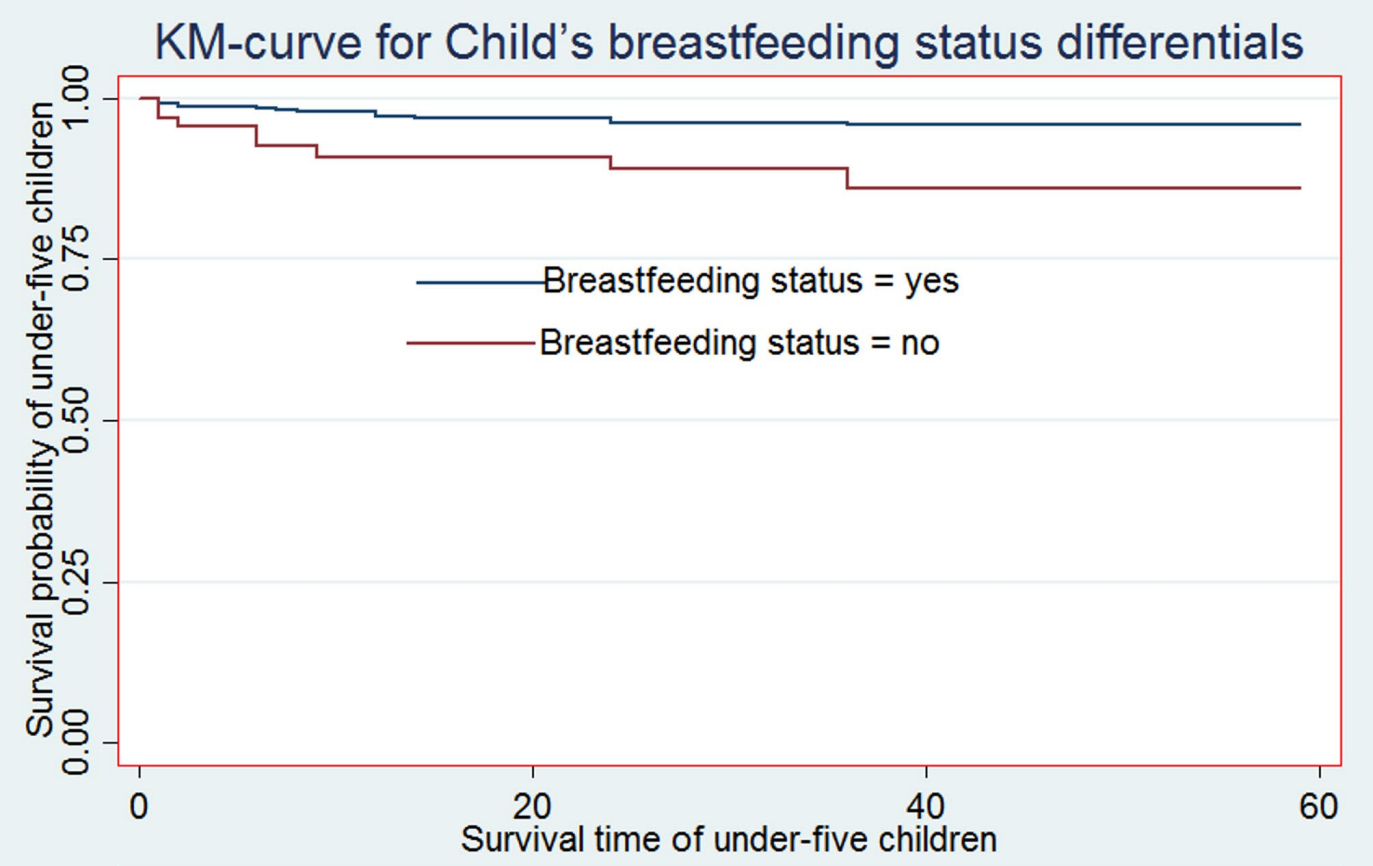
Survival curve by Child’s breastfeeding status

**Fig. 3:**
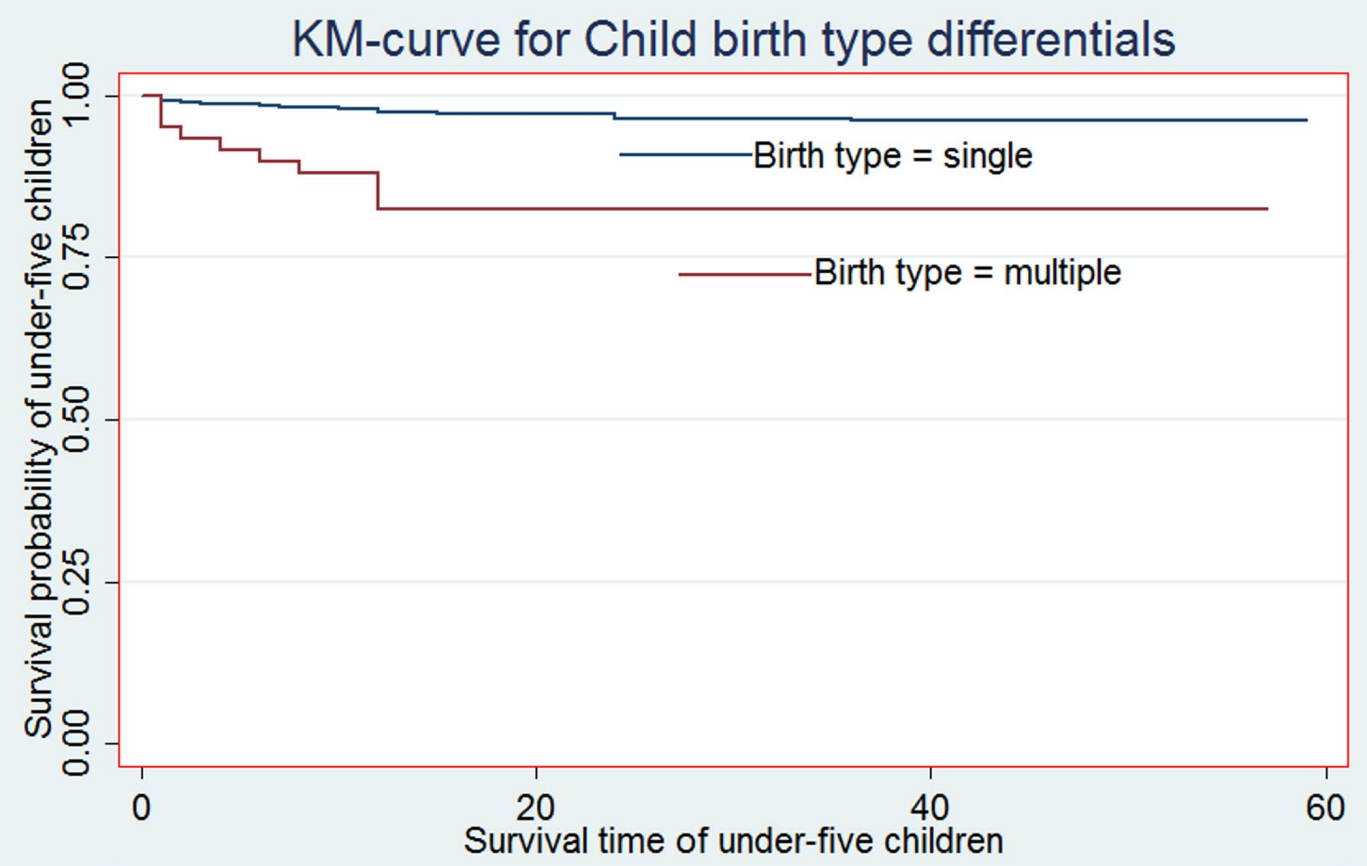
Survival curve by Child’s birth type

**Fig. 4:**
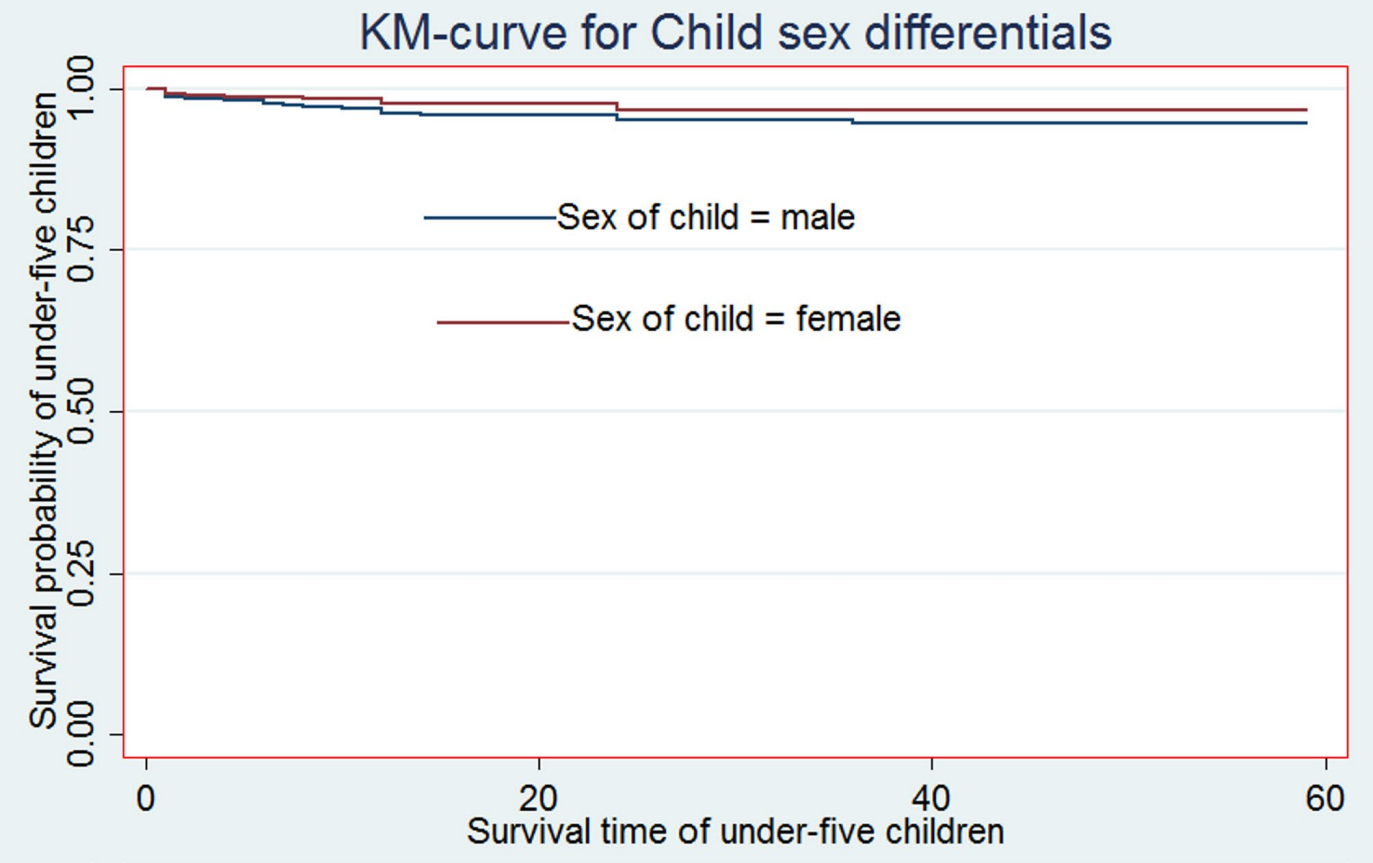
Survival curve by Child’s sex

For the most significant predictor factors, Kaplan-Meier (K-M) non-parametric survival curve analysis estimators are presented in addition to the descriptive data provided in Table 2. According to these Kaplan-Meier (K-M) curves, children who did not breastfeed, have multiple births, and male sex of child have a shorter survival time than those reference categories (see Figs. 2, 3 and 4).

Moreover, from the log-rank test, there was a significant difference between the mother’s ANC follow-up (P-value = 0.001), toilet facility (P-value = 0.0377), sex of household head (P-value = 0.032), parity (P-value = 0.001), sex of the child (P-value = 0.044), birth type (P-value = 0.001), and exclusive breastfeeding (P-value = 0.001) on the time-to-death among under-five children (see Additional File 1: Table S1).

### Predictors of death among under-five children in developing regions of Ethiopia

In crude hazard ratio (CHR), the result showed that multiple births, being non-breastfeeding, sex of household (female), ANC follow-up (no), and sex of the child (female) were significant predictors of under-five children’s mortality (P-value < 0.05). Children who don’t breastfeed are at higher risk of mortality than breastfeeding children (CHR = 3.4, 95% CI: 1.64–7.19). Children whose mothers were not attending ANC follow-up during pregnancy (CHR = 2.5, 95% CI: 1.39–4.54) were at greater risk of mortality than children whose mothers were attending ANC follow-up during pregnancy.

Children who have multiple birth types also had a higher risk of mortality than their counterparts (CHR = 5.4, 95% CI: 2.75–10.55).

From the adjusted hazard ratio (AHR), the results revealed that multiple births, being non-breastfeeding, sex of household (female), ANC follow-up (no), and sex of the child (female) were significant predictors of under-five children’s mortality (P-value < 0.05). The adjusted hazard ratio (AHR) of the mortality rate was 2.9 times higher among under-five children who were of multiple birth type than among those who were singletons (AHR = 2.9; 95% CI: (1.33–6.46)). Similarly, when we compare the mothers ANC follow-up during pregnancy, we find it is statistically associated with the child death rate. The AHR of the child mortality rate was approximately 2 times higher among children who did not attend ANC follow-up during pregnancy than among those who attended ANC follow-up during pregnancy (AHR = 2; 95% CI: 1.08–3.87).

Additionally, an essential demographic factor significantly associated with under-five child mortality was the sex of the child. Being male children were at a higher risk of death. Moreover, for the variable breastfeeding status of children, the AHR of the child death rate of breastfeeding children was approximately 2.7 times higher than that of children who had the non-breastfeeding status of children (AHR = 2.7; 95% CI: 1.23–5.78). The result of the likelihood ratio test (LRT) had 58.43 and a degree of freedom of 15 with a p-value = 0.000, which is statistically significant at the 5% level of significance, and the concordance = 0.81 explained that the dataset and the model were in good fit. Moreover, since the overall global test (p-value = 0.70) for all predictors is > 0.05 and none of the predictor variables failed the Cox PH model, the essential assumption is satisfied. Consequently, the model violated the Cox PH assumptions, and it is mathematically adequate (see Tables 3 and 4).

**Table 3:** Summary of sociodemographic, economic and clinical associated factors of under-five children mortality in the developing regions of Ethiopia, from March 21, 2019, to June 28, 2019 (*N* = 2,019)

Variables	Categories	Censored *N* (%)	Death *N* (%)	Total
Sex of child	Male	991(49.1%)	94(4.7%)	1085(53.7%)
	Female	864(42.8%)	70(3.5%)	1085(46.3%)
Maternal age	45–49	41(2.03%)	15(0.74%)	56(2.77%)
	40–44	101(5.00%)	6(0.30%)	107(5.30%)
	35–39	237(11.74%)	24(1.19%)	261(12.93%)
	30–34	371(18.76%)	30(1.49%)	401(19.86%)
	29 and below	1105(54.73%)	89(4.40%)	1194(59.14%)
Toilet facility	Yes	648(32.10%)	61(3.02%)	709(35.11%)
	No	1207(59.78%)	103(5.10%)	1310(64.88%)
Birth type	Singleton	1803(89.30%)	137(6.79%)	1940(96.09%)
	Multiple	52(2.57%)	27(1.34%)	79(3.91%)
Parity	3 and below	825(40.86%)	65(3.22%)	890(44.08%)
	4–5	457(22.63%)	32(1.59%)	489(24.22%)
	6–8	452(22.39%)	37(1.83%)	489(24.22%)
	9 and above	121(6.00%)	30(1.49%)	151(7.48%)
Birth order	3 and below	931(46.11%)	76(3.76%)	1007(49.87%)
	4–5	448(22.19%)	33(1.63%)	481(23.82%)
	6 and above	476(23.58%)	55(2.72%)	531(26.30%)
Breastfeeding	No	1792(88.77%)	97(4.80%)	1889(93.56%)
	Yes	63(3.12%)	67(3.32%)	130(6.44%)
ANC follow up	Yes	843(41.75%)	41(2.03%)	884(43.78%)
	No	1012(50.12%)	123(6.09%)	1135(56.22%)
Region	Benishangul-Gumuz	485(24.02%)	45(2.23%)	530(26.25%)
	Somali	580(28.73%)	57(2.82%)	637(31.55%)
	Gambela	415(20.56%)	35(1.73%)	450(22.29%)
	Afar	375(18.57%)	27(1.34%)	402(19.91%)
Maternal educational level	Higher	113(5.60%)	4(0.20%)	117(5.80%)
	Secondary	163(8.07%)	6(0.30%)	169(8.37%)
	Primary	538(26.65%)	58(2.87%)	596(29.52%)
	No education	1041(51.56%)	96(4.76%)	1137(56.32%)
Media exposure access	Yes	336(16.64%)	27(1.34%)	363(18%)
	No	1519(75.24%)	137(6.78%)	1656(82.02%)
Wealth index	Rich	496(24.57%)	39(1.93%)	535(26.50%)
	Middle	200(9.90%)	15(0.74%)	215(10.65%)
	Poor	1159(57.40%)	110(5.45%)	1269(62.85%)
Currently pregnant	No	1616(80.04%)	138(6.84%)	1754(86.87%)
	Yes	239(11.84%)	26(1.29%)	256(13.13%)
Current contraceptive method	No	1416(70.13%)	132(6.54%)	1548(76.67%)
	Yes	439(21.74%)	32(1.59%)	55(23.32%)
Marital status	Married	1712(84.80%)	150(7.43%)	1862(92.22%)
	Separated	54(2.68%)	1(0.05%)	55(2.72%)
	Widowed	31(1.54%)	3(0.15%)	34(1.68%)
	Divorced	58(2.87%)	10(0.50%)	68(3.37%)
Place of delivery	With health facility	1795(88.9%)	155(7.68%)	1950(96.58%)
	Home	60(2.97%)	9(0.45%)	69(3.42%)
Delivery by caesarean section	No	1765(87.4%)	157(7.8%)	1922(95.2%)
	Yes	90(4.5%)	7(0.35%)	97(4.8%)
Counseling during pregnancy	Yes	1578(78.2%)	145(7.2%)	1723(85.3%)
	No	277(13.7%)	19(0.94%)	296(14.7%)

**Table 4:** Bivariable and multivariable Cox PH regression model analysis of associated with under-five children mortality in the developing regions of Ethiopia, 2019 (*N* = 2,019)

Variables	CHR (95% CI)	AHR (95% CI)
Sex of child (ref. = male)		
Female	0.6(0.357–0.993) *	0.56(0.333–0.934) *
Maternal age (ref. = 45–49)		
40–44	0.38(0.101–1.45)	0.57 (0.151–2.15)
35–39	0.52(0.186–1.46)	0.62 (0.214–1.82)
30–34	0.29(0.101–0.837) *	0.58 (0.187–1.79)
29 and below	0.31(0.122–0.804) *	0.60(0.190–1.88)
Toilet facility (ref. = yes)		
No	1.8(1.024–3.24)	1.5(0.803–2.67)
Birth type (ref. = single)		
Multiple	5.4(2.75–10.55)	2.9(1.33–6.46) *
Sex of household (ref. = male)		
Female	0.48(0.249–0.954) *	0.47(0.236–0.923) *
Parity (ref. = 3 and below)		
4–5	1.0(0.515–1.95)	0.79(0.292–2.15)
6–8	0.8(0.389–1.64)	0.49(0.091–2.66)
9 and above	4.3(2.34–8.04)	1.7(0.252–10.82)
Birth order (ref. = 3 and below)		
4–5	0.86(0.44–1.69)	1.1(0.343–3.22)
6 and above	1.7(1.01–2.95) *	1.4(0.248–8.20)
Breastfeeding (ref. = no)		
Yes	3.4(1.64–7.19) *	2.7(1.23–5.78) *
ANC follow up (ref. = yes)		
No	2.5(1.39–4.54) *	2.0(1.08–3.87) *
N.B: - CHR: crude hazard ratio, AHR: adjusted hazard ratio, * Significant at 5% level of significance, ref: reference, CI: confidence interval, LRT chi^2^ (15) = 58.43 and Prob > chi^2^ = 0.0000*.		

## Discussion

The overall aim of this study was to study the time-to-death of U5CM and its predictors in the developing regions of the Ethiopia using nationally representative recent standard MDHS data from 2019 by using the Cox Proportional Hazards (PH) regression model analysis. According to this study, the developing regions of Ethiopia overall U5CM rate was 8.1% (95% CI 7.0%, 9.4%). This finding is consistent with other research conducted in Nigeria (104 fatalities per 1,000 total live births), Ethiopia (61 deaths per 1,000 total live births) [3], and Sub-Saharan Africa (SSA) (81 deaths per 1,000 total live births) [8]. The author’s findings, however, are lower than those of other investigations carried out in the Central African Republic (124 deaths per 1,000 total live births), Sierra Leone (114 deaths per 1,000 total live births), Mali (111 deaths per 1,000 total live births), and Somalia (133 deaths per 1,000 total live births) [18].

Moreover, it is higher than the average world index 34 deaths per 1,000 total live births [19] and the study conducted in Somali 57 deaths per 1,000 total live births [20].

There was a substantial correlation established between birth type and under-five child mortality. Compared to singleton birth types, the risks of under-five child mortality were greater for multiple birth types. This aligns with various research investigations carried out in Ethiopia [4, 21, 22]. This may be due to the fact that prematurity and growth retardation, the two primary risk factors for the death of children under five, can result from numerous birth types [23]. Furthermore, having twins increases the risk of under-nutrition from insufficient breast milk and infections from improper cow’s milk feeding.

The sex of the child was one important demographic characteristic linked to U5CM. We also found indication that the lower risk of U5CM was found among female’s under-five children as compared to male children. A related study in the literature suggested that males are more susceptible to illness, infection, and early death, and that this may be caused by genetic differences between male and female children [1, 10, 21, 22, 24].

The study also found that one of the predictive variables linked to U5CM was the child’s breastfeeding status. The risk of death was twice as great for children who didn’t receive breastfeeding from their moms as for those who did. This is in line with the results of a cross-sectional study done in Ethiopia [10], Afar, Ethiopia [25], Southern Ethiopia [26], Ethiopia [27], and rural Ethiopia [28]. The explanation for this could be that a child’s chances of surviving are significantly increased by breastfeeding since the mother’s milk shields them from infection [29]. This means that children who have not been fed in accordance with the recommended breastfeeding timetable are vulnerable, and the likelihood of their surviving will be decreased.

Furthermore, the children of pregnant women who had ANC follow-up were at a lower risk of dying than the children of moms who did not receive ANC follow-up. This result is in line with research done in Ethiopia [5, 30], although it differs from another study [31]. The reason for this could be that moms who make appropriate use of maternal health services, such as ANC follow-up, could provide the mother with pertinent information about the health of her unborn child and appropriate feeding practices to avoid contaminations [32]. Additionally, moms who receive ANC follow-up may be able to access postpartum care, which is essential for looking for potential health issues in their children and implementing the right interventions.

The study found that another significant demographic factor influencing U5CM in Ethiopia’s emerging regions is the head of household being female. Compared to female household heads, male household heads had decreased probabilities of U5CM. Children born to moms with fewer total children ever had the lowest odds of U5CM [8, 22].

### Strengths and limitation of the study

Although the fact that the 2019 EMDHS data collection is a large-scale and nationally representative dataset, this study may have a number of drawbacks. First of all, recall bias could be present because respondents were asked to recollect events from the five years before to the study, during which time some details might have passed. Second, the 2019 EMDHS datasets contained a significant amount of missing datasets. Furthermore, the study excluded a number of important predictor variables, including the age of the pregnant mother.

Thirdly, because the survey’s dataset is cross-sectional, it is challenging to identify the causal links between exposure and response variables. Fourthly, the data is based on self-reported life histories obtained from a nationally representative survey, which is prone to many types of inaccuracy. For example, estimates for individual countries may be exaggerated over time. Finally, the researchers should be informed that multilevel model analysis is a better way to mitigate these limitations when their dataset has a hierarchical structure, as in the 2019 EMDHS datasets.

## Conclusions

The study highlights an important under-five child mortality rate of 8.1% in the developing regions of Ethiopia. Important predictors identified through multivariable Cox Proportional (PH) regression model analysis include multiple birth type, lack of mother’s antenatal care (ANC) follow-up, the child’s sex, gender of household head, and failure to begin exclusive breastfeeding. Therefore, in order to decrease the high rate of mortality among children under five, the government should emphasize improvement of the ANC services, promote exclusive breastfeeding, make a targeted intervention for multiple births, and support female households in the developing regions of Ethiopia.

## Data Availability

The dataset will be shared up on request and will be obtained through contacting emailing to the corresponding author (Gebru Gebremeskel Gebrerufael) using “ gebrugebremeskel2015@gmail.com ”.

## Abbreviations

AHR: Adjusted Hazard Ratio
ANC: Ante-Natal Care
CHR: Crude Hazard Ratio
CI: Confidence Interval
CSA: Central Statistical Agency
EMDHS: Ethiopia Mini Demographic Health Survey
KM: Kaplan-Meier
LRT: Likelihood Ratio Test
PH: Proportional-Hazards
SSA: Sub-Saharan African
U5CM: Under-5 Children Mortality
